# Persistent entropy links irregularities in daily and weekly rest and activity cycles during gestation week 22 and 32 to maternal and neonate health outcomes: A prospective cohort study

**DOI:** 10.1371/journal.pone.0342509

**Published:** 2026-03-16

**Authors:** Sashiel Vagus, Theresa M. Casey, Uduak Z. George

**Affiliations:** 1 Department of Mathematics and Statistics, San Diego State University, San Diego, California, United States of America; 2 Department of Animal Sciences, Purdue University, West Lafayette, Indiana, United States of America; University of Oxford, UNITED KINGDOM OF GREAT BRITAIN AND NORTHERN IRELAND

## Abstract

Though rest (i.e., sleep and periods of inactivity) and activity levels are known to be altered during pregnancy, the effect of day-to-day consistency in rest-activity cycles on maternal-neonate health is not well characterized. Actigraphy objectively measures rest-activity; however, the large datasets it generates often reveal irregular patterns that are challenging to interpret. We introduce a novel approach that applies persistent homology and entropy, quantitative measures in the field of topological data analysis, to quantify disorder in actigraphic data and examine their association with maternal and neonate health. Actigraphic data were collected from a prospective observational cohort study of pregnant women, with observations at gestational weeks 22 (G22; n = 41) and 32 (G32; n = 44). Persistent entropy was computed for daily and weekly actigraphic data. Participants who experienced maternal or neonate complications, including gestational hypertension, preeclampsia, etc., and adverse birth outcomes had higher average daily entropy at G22 and G32, indicating greater temporal disorder in rest-activity. Furthermore, complicated pregnancies had higher odds of exhibiting high entropy compared to uncomplicated pregnancies OR = 10.38, 95% CI [2.03, 74.68]; Fisher’s exact test, p = 0.001), especially in participants with high BMI (OR = 24.00, 95% CI [1.76, 1549.19]; Fisher’s exact test, p = 0.005. Receiver operating characteristic (ROC) analysis showed that weekly entropy at G22 had acceptable predictive power (AUC = 0.70; threshold = 8.24; sensitivity = 0.86; specificity = 0.70), whereas entropy at G32 was a weaker predictor. While the analysis yielded a significant association between high entropy at G22 and complicated pregnancies, the wide 95% CI [1.76, 1549.19] suggests that the magnitude of this effect should be interpreted with caution due to the limited sample size. These findings suggest that persistent entropy may be a useful tool for linking rest-activity irregularities to maternal and neonatal health outcomes. Larger studies are needed to evaluate its potential as a predictive model.

## 1 Introduction

Maternal health complications adversely affect the health and mortality of neonates [1–6]. In the United States, maternal morbidity and mortality continue to increase, nearly tripling between 1990 and 2019 [7], with the rates in the United States higher than other developed nation [8,9]. Each year, about 60,000 women in the United States experience life-threatening maternal morbidity, leading to more than 700 pregnancy-related deaths [8], with the primary cause being high blood pressure related morbidities during pregnancy (i.e., gestational hypertension, pre-eclampsia, and eclampsia) [10]. Alarmingly, pregnancy complications may lead to lifelong sequelae, affecting the woman‘s health and the health of children exposed to the condition in utero [11].

Day-to-day consistency in the timing of sleep, wake, and physical activity plays a substantial role in maintaining good health [12,13]. The amount and regularity of physical activity and sleep can influence physiological and neurobehavioral health [14], and are linked to circadian clock disruption [15]. Disruptions in the circadian system during gestation may contribute to various adverse maternal and neonate health outcomes [16–20], including miscarriage [19,21], gestational diabetes mellitus [22], preeclampsia [23,24], and an increased risk of preterm birth [25]. Although sleep and physical activity are known to be altered during pregnancy [26], the effect of the day-to-day consistency and weekly consistency in physical activity and sleep-wake cycles on maternal-neonate health is not well characterized.

Wrist actigraphy provides a non-invasive and objective way to measure movement, and is essential for understanding how physical activity and sleep quality influence health outcomes. Actigraphy data reflects patterns of rest (i.e., sleep and inactivity) and activity (i.e., physical activity). Analysis of actigraphy data enables inference of the consistency and timing of physical activity and sleep-wake cycles based on the magnitude of activity counts, with a general assumption of different movement intensity in each [27–30]. Specialized algorithms in computer software programs have been used to estimate sleep parameters from recorded wrist actigraphy such as sleep duration, sleep fragmentation index, timing of sleep onset, and sleep efficiency and wake after sleep onset [31,32]. Actigraphy measures of sleep have yielded insight regarding the importance of the amount of sleep and patterns of sleep during gestation on maternal and neonate health [33–36]. Wrist actigraphy data also captures patterns of activity such as frequency, duration, intensity and timing [35]. However, very few studies have quantified how daily or weekly irregularities in the amount and timing of rest and activity (i.e., the cyclic pattern and shape of the rest-activity data) associate with maternal health, despite research indicating its potential importance [37].

Actigraphy data are large time-series data that contain irregular visual patterns, making interpretation difficult. Previous methods applied for abstracting the amount and timing of rest-activity patterns from actigraphic data include fast Fourier transform [38,39], cosinor analysis [40], and root mean square successive difference (RMSSD) [36]. However, these approaches have some drawbacks. Though fast Fourier transform is computationally inexpensive and simple [41], the length of the data segments used in the analysis affects the oscillation and frequency resolution. Therefore, artificial interpolation is often used to satisfy the need for equal data segments. However, the artificial interpolation of the data introduces bias. Cosinor analysis involves fitting a cosine function to time-series data to estimate rhythm characteristics like amplitude, mesor, and acrophase [42]. A drawback of cosinor analysis is that it cannot measure random fluctuations [43], which are a common characteristic of rest-activity data. Moreover, if the rest-activity data oscillates with varying amplitudes, then the cosine function will not adequately capture the rhythmic patterns in the data [44]. RMSSD has been used to measure the degree of variability in rest-activity data, and subsequently correlated with maternal health [36]. A limitation of RMSSD is that it is sensitive to artifacts [45], meaning even a single outlier or a short interval of abnormal rest-activity can significantly impact its calculated value. Therefore, novel approaches for analyzing irregularities in the amount and timing of rest and activity in actigraphic data are needed.

This study aimed to determine if the amount and timing of daily and weekly rest and activity during gestation week 22 (G22) and G32 is associated with maternal and neonatal health outcomes. We hypothesized that greater inconsistencies in the amount and timing of rest and activity would increase the risk for poorer health outcomes. Gestational time points selected aimed to capture distinct metabolic states of gestation, with G22 occurring during the anabolic phase of pregnancy and G32 the catabolic phase [46–49]. The anabolic phase occurs during the first and second trimesters of pregnancy, where there is an increase in maternal lipid synthesis and fat storage, in preparation for the increases in energy needed to support fetal growth in late pregnancy and milk production during lactation [50,51]. Conversely, during the third trimester, lipid metabolism enters a catabolic phase characterized by the breaking down of maternal fat stores to supply essential substrates required for fetal growth and development [50,51]. To quantify the inconsistencies in the amount and timing of daily and weekly rest and activity during G22 and G32, we introduce persistent entropy as a novel approach for interpreting actigraphic data. A computational tool from the field of topological data analysis (TDA), persistent entropy is capable of capturing the topology of the temporal data. We reasoned that persistent entropy can condense irregular or inconsistent rest-activity patterns during gestation into quantifiable indicators that can be used to examine how rest-activity relate to maternal and neonatal health outcomes.

Although TDA is computationally intensive to implement for large datasets, a major advantage of TDA over existing methods is that it evaluates inconsistencies in the timing and amount of rest and activity over a given period by analyzing the shape/topology of the rest-activity data. Therefore, it performs well even when the data contains rhythm fragmentation or random fluctuations, compared to other actigraphy-analysis methods. In other words, the key strength of TDA is that it accurately characterizes irregularities in rest-activity data regardless of rhythm fragmentation or random fluctuations. Furthermore, we compared findings from TDA to cosinor analysis to determine the performance of both methods in predicting maternal and neonate health. We demonstrate that by combining the outputs from TDA and cosinor analysis, we can enhance our understanding of how rest-activity at G22 and G32 influence pregnancy outcomes. Testing this approach in a large sample size and in other populations is essential to confirm its effectiveness and to determine its generalizability and robustness.

The rest of the article provides a detailed description of the study methodology, including topological data analysis, the participant selection criteria, data collection methods, ethical considerations and a novel framework for modeling rest-activity with persistent entropy. The findings from the study are presented in the results section and further interpreted in the Discussion and Conclusion.

## 2 Methodology

### 2.1 Topological data analysis

**Fig 1:**
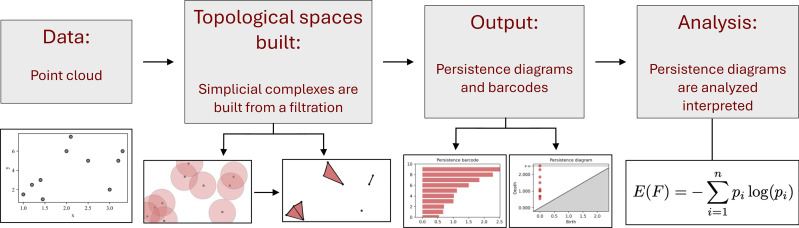
Key steps for implementing topological data analysis. The process begins with a point cloud, followed by the construction of a topological space using a filtration F.  The persistence diagram of the topological space is then analyzed using persistent entropy E(F.

TDA allows the rest-activity data to be studied through persistent homology [52] (Fig 1). Persistent homology is a mathematical technique used in the field of algebraic topology and computational geometry, in which qualitative features are taken into consideration for their persistence in different scales [53,54]. This type of homology involves a filtration of a dataset. As the filtration parameter changes, one can compute the homology at different values of that parameter. This is done by creating a filtration of a simplicial complex, which consists of nested sequences of increasing subsets (i.e., building a series of increasingly complex structures from simpler ones) [55]. A filtered simplicial complex K is defined as a collection of subcomplexes {K(t):t∈ℝ}  of *K* such that K(t ⊂  K(s for t<s.  This means that as *t* increases, the subcomplex K(t grows or remains the same. And there exists a maximum filtration time $t_{max}\;$ ∈  ℝ  such that $K_{t_{max}}=K\;$. This means that at $t=t_{max},\;$ the subcomplex $K_{t_{max}}\;$ contains all the simplices of K.  The filtration time (or filter value) of a simplex *σ* ∈  *K* is the smallest *t* such that *σ* ∈  K(t. Depending on the dataset, this method can help the user determine if the data is noisy and identify which features are important. TDA is commonly used to compare datasets. The key steps for TDA are outlined in Fig 1. TDA has been used for the analysis of data from many different areas, including the study of cancer to identify mutations [56], the connectivity of neurons in the brain [57], as well as the spread of contagious diseases [58].

#### 2.1.1 Vietoris-Rips filtration.

**Fig 2:**
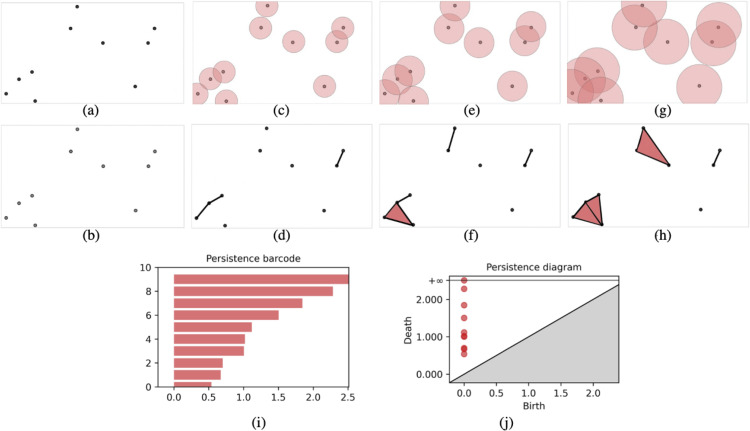
Vietoris-Rips filtration. In **(a)**, **(c)**, **(e)** and **(g)**, the red circles show the increasing radius of the loops and the corresponding simplicial complex is created below the images in **(b)**, **(d)**, **(f)** and **(h)** respectively. As the radius increases, the simplicial complex begins to develop from points, then to line segments which are also known as edges, and then to triangles. **(i)** is the persistence barcode. It quantifies the Vietoris-Rips complex using horizontal bars. **(j)** is the corresponding persistence diagram. The persistence diagram quantifies the Vietoris-Rips complex using dots. Each dot represents, on the y-axis, the *ϵ* value at which a topological feature ceased to exist, and on the x-axis, the *ϵ* value at which the feature was born. Observe that $\epsilon_{birth}=0\;$ for the point cloud in **(a)**.

A commonly used filtration technique is the Vietoris-Rips filtration. The Vietoris-Rips filtration is a process of building a nested collection of Vietoris-Rips complexes on a metric space by increasing a scale parameter [59]. Let *X* ⊂  ${\mathbb{R}}^{D}\;$ be a point cloud and *ϵ* ≥ *0*, then the Vietoris-Rips complex $VR_{\epsilon }\;$ at scale *ϵ* is defined as $VR_{\epsilon }(X)=\;$ {σ⊆X  ∣∀u,v  ∈  σ,  d(u,v ≤ 2ϵ} . In other words, the Vietoris-Rips complex of a point cloud consists of all those simplices whose vertices are at pairwise distance d(u,v no more than 2ϵ.  For a given scale parameter ϵ≥0,  each point u∈X  is a vertex in the Vietoris-Rips complex. An edge is added between two vertices *u* and *v* if the distance d(u,v)≤2ϵ.  A p-simplex *σ* = $[u_{0},u_{1},\ldots ,u_{p}]\;$ is added to the complex if the distance $d(u_{i},u_{j}$ ≤ 2*ϵ* for all pairs $(u_{i},u_{j}).\;$ In other words, a p-simplex is added if every pair of its vertices is within distance 2*ϵ* [60]. This results in a simplicial complex shown in Fig 2.

By tracking these events, one can create a persistence diagram that records the birth and death of topological features, such as connected components (0-dimensional), loops (1-dimensional), and voids (2-dimensional and higher), within the point cloud. Each point in a persistence diagram represents a feature (connected component, loop, void) with coordinates $(\epsilon_{birth},\epsilon_{death}$, where $\epsilon_{birth}\;$ is the value of *ϵ* when a topological feature is first formed and $\epsilon_{death}\;$ is the value of *ϵ* when the topological feature ceases to exist. Features near the diagonal $(\epsilon_{birth}=\epsilon_{death}$ are short-lived and considered noise. Features far from the diagonal are persistent and considered significant. This approach provides insights into significant topological features and their persistence, distinguishing between noise and meaningful patterns in the data. It helps in understanding the topological structure of the data across different scales of *ε*.

In this study, we focused on dimension 0 for simplicity and also because the persistence diagrams in dimension 0 were found to depict the important features of the data effectively, compared to higher dimensions. The birth of 0-dimensional features occurs when each point is initially considered as an isolated component. The death of 0-dimensional features occurs when these components merge as the scale parameter *ϵ* increases (Fig 2j). Persistence diagrams are stable. That is, small changes in the input data (point clouds) lead to small changes in the output (persistence diagrams) [61]. It is important to have these stability properties for real-world problems because if small perturbations are creating big discrepancies, then the results would not be accurate. In other words, stability is required for reliable predictions.

#### 2.1.2 Persistent entropy.

One of the final steps for TDA is to interpret the results from the persistence diagrams. This is accomplished by using persistent entropy. The persistence diagram‘s entropy is an extension of the main concepts from Shannon entropy. Shannon entropy quantifies the uncertainty or the amount of information within a dataset. For a dataset with probability distribution: $p=\{p_{1},...,p_{n}\},\;$ where $p_{i}\;$ is the probability of the *i*-th event occurring, Shannon entropy H(p = -$\sum_{i=1}^{n}p_{i}\log_{2}(p_{i})=\sum_{i=1}^{n}p_{i}\log_{2}\frac{1}{p_{i}}.\;$ When $p_{i}=0\;$, $p_{i}\log_{2}(\frac{1}{p_{i}}$ is set to 0 [62]. A high entropy indicates that the dataset contains a high level of unpredictability, and a low entropy indicates that the dataset is more predictable or concentrated around fewer outcomes. In order to apply entropy to persistent homology, the formula was modified to obtain the persistent entropy formula [63]. Given a filtration *F* = {K(t |  t∈ℝ}  and the corresponding persistence diagram $dgm(F)=\{a_{i}=(x_{i},y_{i}$ | 1≤i≤n},  where $x_{i}<y_{i}\;$ for all *i* and $\{(x_{i},y_{i}\}\;$ represent the multiset of birth and death pairs of topological features, let L=  $\{l_{i}=y_{i}-x_{i}\;$ |  1≤i≤n}.  The persistent entropy E(F of *F* is calculated as follows [55]: E(F = -$\sum_{i=1}^{n}p_{i}\log(p_{i}$ where $p_{i}\;$ = $\frac{l_{i}}{S_{L}},\;$ and $S_{L}=\sum_{i=1}^{n}l_{i}\;$. The persistent entropy formula measures the variability in the lengths of the bars in a persistence barcode (Fig 2i).

**Fig 3:**
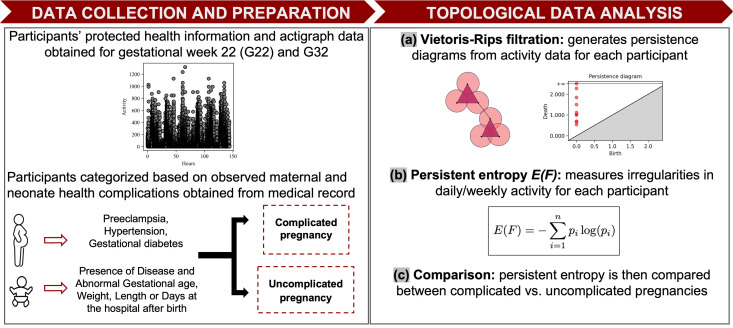
Study design: relating pregnancy outcomes to persistent entropy of participant’s rest-activity.

Persistence diagrams and persistence barcodes represent the same information but with different visual representations. Persistence barcode is a graphical representation of the birth and death of topological features, depicted as bars (Fig 2i). Each topological feature is represented by a horizontal bar. The left end and right end of the bar represent the birth and death of a feature respectively. The length of the bar represents the length of the lifespan of a topological feature and indicates the persistence of the feature. The entropy of a persistence barcode can be used to quantify the variability or complexity of the topological features present in the data. A persistence barcode with bars of uniform lengths has small entropy. However, the more varied the lengths of the bars, the greater the entropy of the persistence barcode. Persistent entropy values can be used to compare two separate groups of datasets. Persistent entropy was used by Ruccoa et al. [64] to compare good versus faulty motors. They found that motors with an entropy value greater than 7.5 were more likely to be good motors [64]. Therefore, by using persistent entropy, they were able to determine what level of entropy would lead to optimal results. In this study, entropy played a similar role. We examined whether high entropy values predicted the likelihood of a woman to experience pregnancy complications (Fig 3). However, instead of looking only at which high entropy values relate to undesirable outcomes, we also explored other parameters, including how variability in entropy values over the days in G22 and G32 are related to pregnancy outcomes.

### 2.2 Participant inclusion criteria, data collection methods, and ethical considerations

#### 2.2.1 Demographics of the study population.

This study employed secondary analysis of publicly available de-identified data from a cohort study, obtained from the Purdue University Research Repository [65]. The dataset we analyzed was collected in a prospective observational cohort study conducted from August 1, 2014 to October 17, 2015 at Indiana University, Eskenazi Health and Purdue University. The study was approved by the Institutional Review Board (IRB; protocol number 1405014885) at Indiana University. The primiparous women who participated in this study were between the ages of 18 and 40 years of age, had a single fetus, and were at less than 22 weeks of gestation at the time of recruitment. Participants who completed the study had a median age of 23 years and two-thirds of them were Black/African American, a demographic that is disproportionately affected by poor maternal and neonatal health outcomes. Of the 92 women who were eligible to participate in the study, 50 participated in G22 and 46 participated in G32. The reason for half of the withdrawals from the study were due to the loss of a fetus, relocation, or other life events. Some participants were withdrawn from the study by research staff because of non-compliance with the study protocol. However, no differences in demographic variables between recruited, retained and withdrawn populations. The majority of these women were taken from an economically disadvantaged population (details in S1 Table in S1 Text, [36,42,65]).

#### 2.2.2 Data collection: health information and actigraphic data.

The publicly available de-identified dataset used in our study [65] contained actigraphic data that were obtained from a wrist actigraphy device (Actiwatch Spectrum, Philips Respironics Andover, MA) worn by participants during G22 and G32. Wrist actigraphy is a compact, watch-like device that records data on wrist movement [66,67]. It contains a battery, memory storage, and an accelerometer, which tracks and records movements of the wrist [27]. Each wrist movement is referred to as an activity. Wrist actigraphy data were collected in 30 sec epochs for 3–7 days in the corresponding weeks. To ensure sufficient data for the analysis of rest-activity patterns, only participants with a minimum of six days of continuous actigraphic recordings were included in the current study. The study sample comprised 41 women at G22 and 44 women at G32, following the exclusion of participants with fewer than six days of wrist actigraphy data. All the participants provided written consent for the use of their actigraphic data.

The publicly available de-identified dataset used in our study [65] also contained demographic data and health information. A survey was used to obtain demographic data, once verbal and written consent were received. The participants provided written consent to abstract protected health information (PHI) from their medical record including pre-pregnancy body mass index (BMI), and diagnosis of hypertension, preeclampsia and gestational diabetes mellitus (GDM). The participants also provided written consent to abstract the medical records for their infant including the gestational age at the time of birth, birth weight, birth length, presence or absence of disease and total days of hospitalization after delivery.

#### 2.2.3 Maternal and infant health outcomes: complicated vs. uncomplicated.

For the current study, participants were categorized into one of two groups: complicated (n = 21 for G22; n = 21 for G32) or uncomplicated pregnancy (n = 20 for G22; n = 23 for G32). A pregnancy was deemed complicated if the participant was diagnosed with preeclampsia, hypertension, and/or gestational diabetes. Additionally, a pregnancy was considered complicated if the neonate had any of the following conditions: abnormal birth weight, abnormal birth length, abnormal gestational age, any disease, or a prolonged hospital stay after birth. Abnormal neonatal outcomes were defined based on the following criteria: birth weight outside the range of 2.5–4.0 kg [68,69], birth length outside the range of 47–53 cm [70], gestational age outside the range of 38–42 weeks [71–73], or a hospital discharge occurring more than 4 days after birth [74–76] (S2 Table in S1 Text).

### 2.3 Modeling rest-activity during pregnancy with persistent entropy

#### 2.3.1 Resampling of actigraphic data using Greedy Permutation.

We used scatter plots to visualize daily and weekly temporal trends in rest-activity for each participant in the study. The dataset was large because the actigraphic data were collected in 30-sec epochs, with over 17,000 data points per week for each participant. Some participants had gaps in their data (i.e., missing values) due to removing their wrist actigraphy devices for short or extended periods. Computing the persistence diagram and entropy for each woman using the full dataset was computationally expensive. Therefore, we resampled the actigraphic data using a Greedy Permutation algorithm, reducing it to 8,640 points per week for the weekly TDA analysis and 2,000 points for the daily TDA analysis [77,78]. This reduction cut the data to about half its original size without significant changes in TDA results. We tested the reduction through trial and error to balance computational cost with result accuracy. The Greedy Permutation method was selected to retain the overall shape and key features of the data, making it easier to compute the persistence diagram and entropy for each participant while significantly reducing computational time.

After resampling the data with the Greedy Permutation algorithm, we computed the daily and weekly persistence diagrams and entropy for each participant. We then compared the weekly persistent entropy values between the complicated and uncomplicated pregnancy groups. Additionally, we assessed the daily variability in entropy values for each participant and compared these values between the two groups. Statistical tests to identify differences between the complicated and uncomplicated pregnancy groups were conducted using the Fisher’s exact test and the Mann-Whitney U test.

A one-tailed Mann-Whitney U test was implemented because it was expected that participants with complicated pregnancies would exhibit higher average daily entropy, as there is evidence to suggest that irregular rest-activity patterns are associated with unfavorable pregnancy outcomes [79]. Moreover, night and irregular shift work during gestation have been found to be associated with adverse pregnancy outcomes [80,81]. Therefore, a one-tailed test was applied as the statistical approach to test whether there was a significant (p <  0.05) or trend (0.05 <  p <  0.1) for an increase in entropy in complicated pregnancies.

#### 2.3.2 Calculating the persistent entropy for rest-activity data.

After resampling the data points using the Greedy Permutation algorithm, we used the resampled data to obtain Vietoris-Rips filtrations and persistence diagrams. These were computed using the *Ripser Python package* [82]. Persistence diagrams were generated for both daily and weekly rest-activity data for each participant in groups G22 and G32. After obtaining the persistence diagrams, we calculated the entropy values for each participant on a daily basis for groups G22 and G32, as well as for the entire duration of each group. The entropy values for each persistence diagram were computed using the persistent entropy formula, implemented via the *Persim Python package* [77]. These persistent entropy values were then stored for subsequent statistical analysis.

#### 2.3.3 Calculating the daily variability and the average daily entropy for rest activity data.

The average daily entropy for each participant‘s rest-activity was computed for groups G22 and G32 using the root mean square (RMS) formula. RMS is commonly used to measure variability between samples. The RMS formula is given by: RMS = $\sqrt{(x(1{)}^{2}+x(2{)}^{2}+...+x(n{)}^{2})/n}\;$, where x(i denotes the persistent entropy for a given participant at time *i* and *n* represents the number of days for which the daily entropy was calculated in G22 or G32. The variability in daily entropy was computed for each participant using median absolute deviation (MAD). MAD is often preferred over other measures of variability in samples that may have outliers. The formula for MAD is: $\mathrm{MAD}=\mathrm{median}(|Y_{i}-\tilde{Y}|)\;$, where $Y_{i}\;$ is the daily persistent entropy values, and $\tilde{Y}=\mathrm{median}(Y_{i}$.

#### 2.3.4 Cosinor analysis of rest-activity data.

Cosinor analysis involved fitting a cosine function f(t)=M+Acos(2π(t−ϕ)/T to rest-activity data to determine the characteristics of rhythmic patterns in the data, such as the amplitude (A), period (T), midline estimating statistic of the rhythm (MESOR) and acrophase (*ϕ*), where MESOR (*M*) is the average level around which the data oscillates and acrophase is the timing of the peak within each cycle [40,42].

## 3 Results

### 3.1 Greedy Permutation resampling preserves the shape and visual characteristics of the original rest-activity data

**Fig 4:**
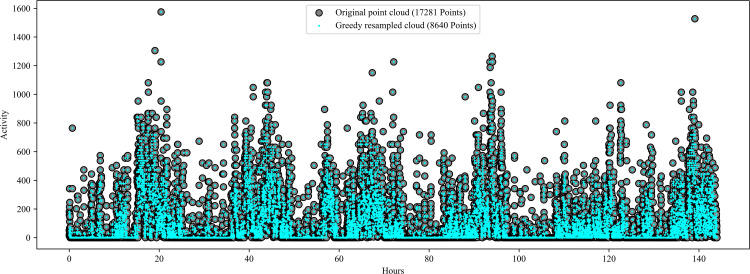
Greedy Permutation Re-sampling. The original point cloud, also referred to as a scatter plot, is displayed alongside the resampled point cloud obtained via Greedy Permutation for the G22 actigraphic data of a single study participant. The y-axis displays the number of activity counts per epoch, while the x-axis represents time. The original point cloud has 17,281 points and is represented by black dots. The resampled data obtained from Greedy Permutation has 8640 points and is represented by dots colored in cyan. Overall, the greedy resample maintains the shape of the data while reducing the number of data points so that the data is more manageable.

**Fig 5:**
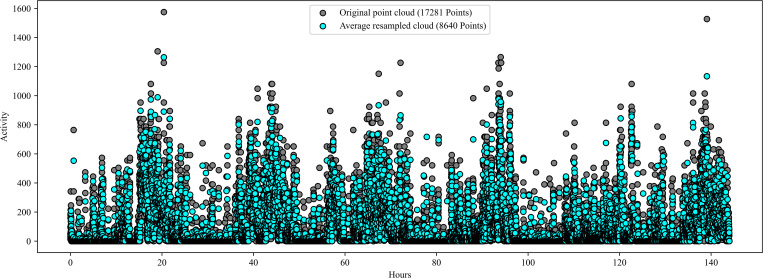
The original point cloud (black dots) is displayed with the resampled point cloud obtained by taking the average of every two successive points (cyan dots). The y-axis displays the number of activity counts per epoch, while the x-axis represents time. Even though we were able to reduce the number of points by half, it is clear that there are many differences between the two point clouds. By performing the average resampling, the shape of the data is changed drastically and many features are lost.

Fig 4 shows a participant’s rest-activity data before and after resampling, demonstrating the effectiveness of the Greedy Permutation algorithm in preserving the essential visual characteristics of the original data. We explored other resampling methods, but none matched the performance of the Greedy Permutation approach. For instance, Fig 5 shows a dataset resampled by averaging every two successive data points, which reduces the data size by half. This method significantly alters the shape of the data, leading to the loss of crucial features. Such alterations can be detrimental to TDA analysis, which depends on preserving the shape and persistent features of the data. After resampling the data with the Greedy Permutation algorithm, we computed the daily and weekly persistence diagrams and entropy for each participant.

### 3.2 Average daily entropy of rest-activity and complicated pregnancy

**Fig 6:**
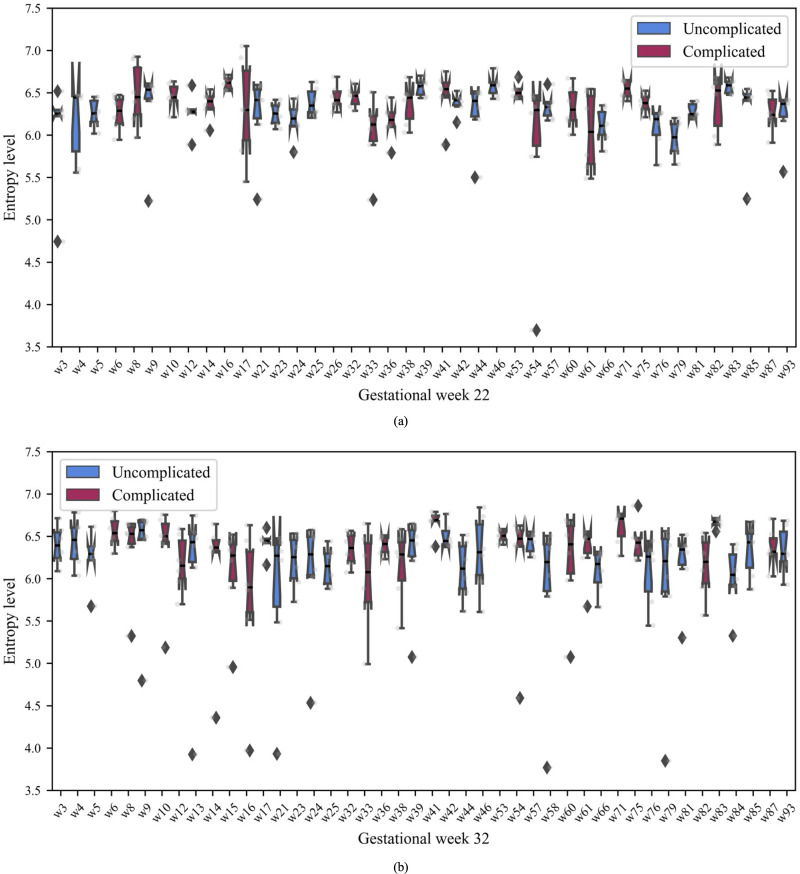
Daily entropy values for G22 and G32. The distribution of daily entropy values in **(a)** G22 and **(b)** G32 participants. The x-axis denotes the participants in the study and the y-axis denotes the daily entropy value. Diamonds represent the outliers within a participants. Some of the participants had very low entropy on one of the 6 days.

The entropy value for each participant’s rest-activity data was computed for each day separately and also for the entire six-day period at G22 and G32. The re-sampled rest-activity data for a 24-hour duration, representing one day of the week, contained 2,000 points. The entropy value for the rest-activity was computed for each day, which we refer to as the daily entropy value. For the six-day period, which consisted of 144 hours, the resampled data included 8,640 points. These data were used to estimate the weekly entropy value for each participant’s rest activity. Daily entropy values for each participant are shown in Fig 6. These plots display the distribution of daily entropy values across the six days for participants in G22 and G32, respectively. In Fig 6a, the box plot for daily entropy values in G22 shows a wider inter-quartile range for participants with complicated pregnancies compared to those with uncomplicated pregnancies.

**Fig 7:**
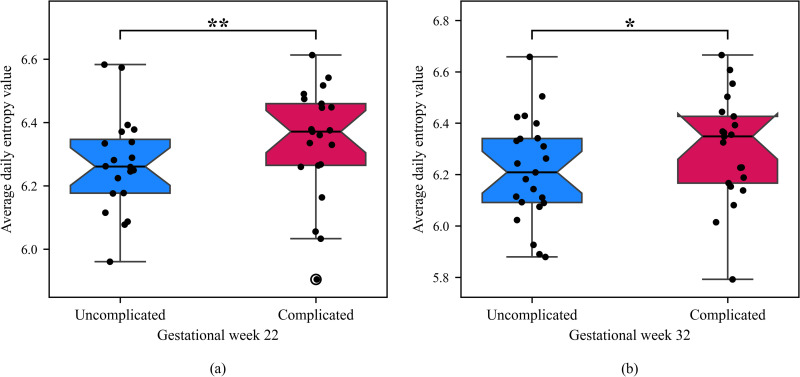
Average daily entropy for (a) G22 and (b) G32. The average daily entropy was computed using the RMS formula. One tail Mann Whitney test indicated that average daily entropy significantly increased in the participants with complicated pregnancies during G22 (p< 0.05, **) and tended to increase during G32 (p <  0.1, *). Circle indicates an outlier.

We examined the relationship between the average entropy of rest-activity and pregnancy outcomes (Fig 7). During G22, the average daily entropy was generally higher for participants with complicated pregnancies compared to those with uncomplicated pregnancies (p = 0.048 for one-tailed Mann Whitney U test; Fig 7a). Similarly, for G32, participants with complicated pregnancies exhibited higher average daily entropy compared to those with uncomplicated pregnancies (p = 0.067 for the one-tailed Mann-Whitney U test; Fig 7b). This indicates that the degree of irregularity in daily rest and activity was on average higher for women in the complicated pregnancy group.

### 3.3 Variability in daily entropy for rest-activity and complicated pregnancies

**Fig 8:**
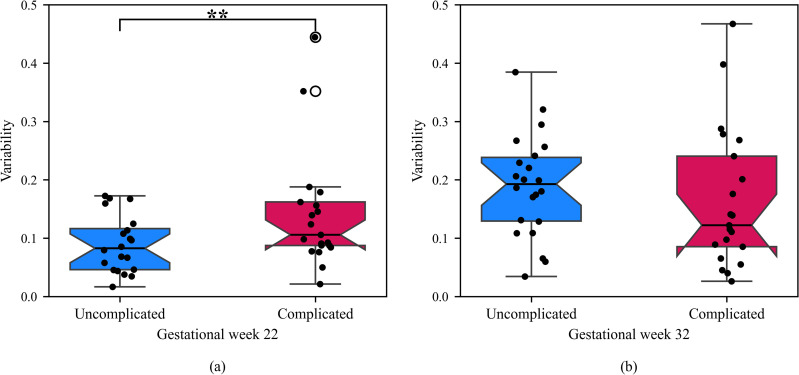
Variability in daily entropy for (a) G22 and (b) G32. Circle indicates an outlier.

The MAD formula was used to compute the degree of variability for each participant’s daily entropy for rest-activity over the six days in G22 and G32. We examined if the variability in the participants’ daily entropy for rest-activity was associated with pregnancy outcomes by comparing the variability for participants in the complicated pregnancy group to those in the uncomplicated pregnancy group during G22 and G32. The box plots for entropy in G22 reveal significant variability in daily entropy for rest-activity in participants with complicated pregnancies compared to those with uncomplicated pregnancies (one-tailed Mann Whitney U test: p = 0.028; Fig 8).

We found no significant difference in the variability of daily entropy for rest-activity between participants with complicated and uncomplicated pregnancies in G32 (one-tailed Mann Whitney U test: p = 0.88). However, notably the variability in daily entropy was significantly higher (p <  0.05) for participants in the uncomplicated pregnancy group in G32 compared to G22, indicating as a normal pregnancy progresses, entropy increases.

### 3.4 Relationship of weekly persistent entropy for rest-activity with pregnancy outcomes

**Fig 9:**
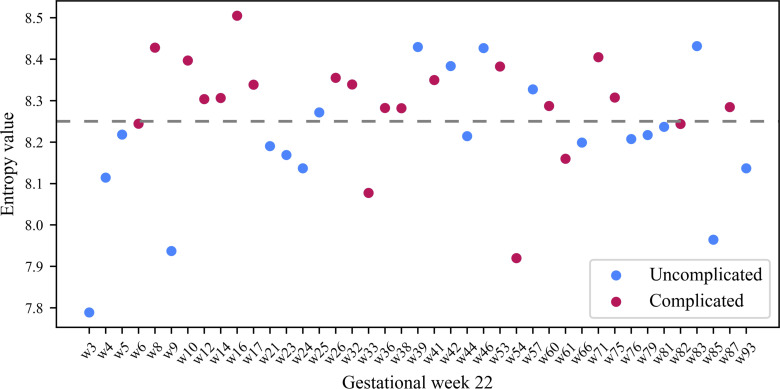
Weekly persistent entropy for rest-activity for participants in G22. The x-axis represents the participants in the study and the y-axis represents their corresponding persistent entropy. The dashed line denotes an entropy value of 8.22.

**Fig 10:**
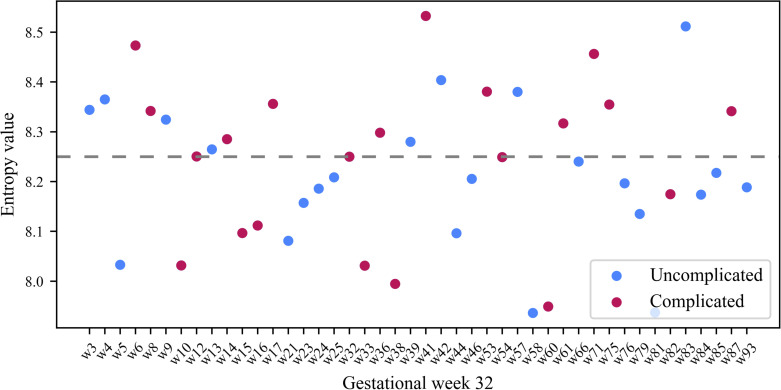
Weekly persistent entropy for rest-activity for G32. The x-axis represents the participants in the study and the y-axis represents their corresponding persistent entropy. The dashed line denotes an entropy value of 8.22.

In addition to analyzing the daily entropy values for the rest activity, we also analyzed the entropy values for weekly G22 and G32 rest-activity for each participant. Figs 9 and 10 show the entropy values for the rest activity for each woman for G22 and G32 respectively. Visually, we see that for G22 (Fig 9), the persistent entropy of women in the complicated pregnancy group were found at a greater frequency above an entropy value of 8.22. In G22, complicated pregnancies were associated with higher odds of high entropy (i.e., > 8.22) compared to uncomplicated pregnancies (OR = 10.38, 95% CI [2.03, 74.68]; Fisher’s exact test, p = 0.001). Although the confidence interval was wide, reflecting small sample size and sparse cell counts, the direction and strength of the association were consistent.

**Fig 11:**
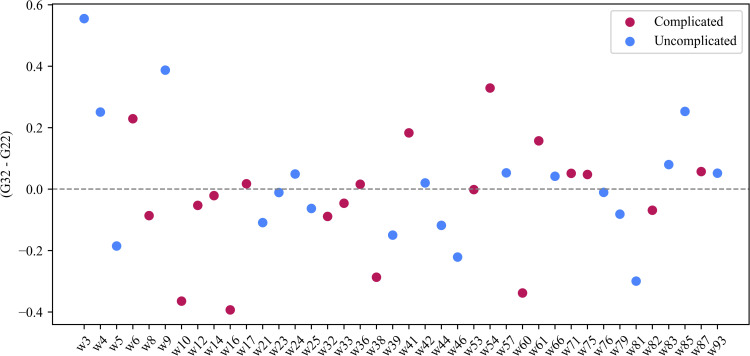
Differences in rest-activity persistent entropy between G32 and G22. The x-axis represents the participants in the study, and the y-axis represents the difference in persistent entropy between G32 and G22.

In G32, complicated pregnancies were associated with higher odds of high entropy compared to uncomplicated pregnancies; however, this association was not statistically significant (OR = 3.03, 95% CI [0.78, 12.86]; Fisher’s exact test, p = 0.080). Two-thirds of the participants with complicated pregnancies in G32 had persistent entropy above 8.22, while about two-fifths of those with uncomplicated pregnancies were above 8.22. We calculated the weekly difference in persistent entropy between G22 and G32 for each participant. Approximately half of the participants exhibited higher persistent entropy at G32 compared to G22, with no observed association with pregnancy complications (Fig 11).

To evaluate whether G22 or G32 weekly entropy predicted pregnancy complications, receiver operating characteristic (ROC) curves were computed and discriminatory performance was quantified using the area under the ROC curve (AUC). ROC analysis showed that weekly entropy during G22 demonstrated acceptable predictive performance; it flagged most complicated pregnancies but mislabeled a few healthy ones (AUC = 0.70; threshold = 8.24; sensitivity = 0.86; specificity = 0.70). On the other hand, weekly entropy at G32 was a weaker predictor of complications (AUC = 0.59; threshold = 8.25; sensitivity = 0.67; specificity = 0.65).

### 3.5 Irregularities in G22 rest-activity are associated with complicated pregnancies irrespective of the participants’ pre-pregnancy BMI

**Fig 12:**
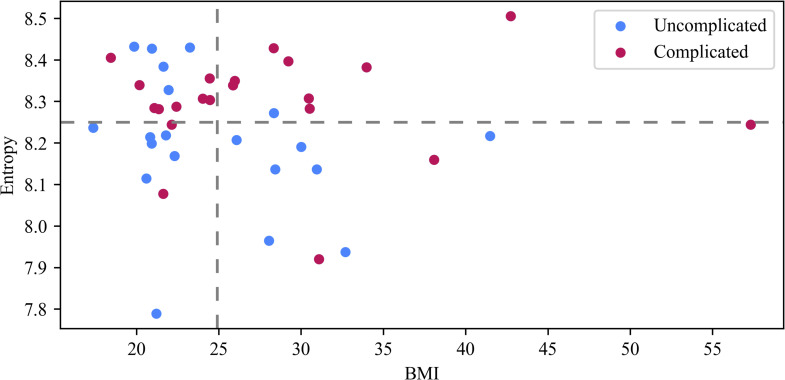
G22 entropy values and BMI. The vertical dashed line (i.e., BMI = 25), separates healthy versus unhealthy BMI. The horizontal dashed line represents an entropy value of 8.22. The unit for BMI is kg/m^2^.

High BMI is often linked to poorer maternal health outcomes [83,84]. Participants were assigned to high BMI (BMI > 25; overweight and obese) and low BMI groups (17.3 <  BMI <  25). We referred to all participants with low BMI as having a healthy BMI because all of them had a BMI in the range of 18.5 <  BMI <  25, except for one participant who had a BMI of 17.3. The weekly persistent entropy for participants in G22 was compared between these groups (Fig 12). Participants were grouped based on their BMI and persistent entropy as follows: low BMI and low entropy, low BMI and high entropy, high BMI and low entropy, and high BMI and high entropy (Fig 12). In particular, we compared participants with high BMI and high entropy to those with high BMI and low entropy (top rightmost quarter versus bottom rightmost quarter, Fig 12). Among participants with high BMI in G22, complicated pregnancies had substantially higher odds of high entropy compared with uncomplicated pregnancies (OR = 24.00, 95% CI [1.76, 1549.19]; Fisher’s exact test, p = 0.005). While the analysis yielded a significant association (OR = 24.00, p = 0.005), the wide 95% CI [1.76, 1549.19] indicates that the magnitude of this effect should be interpreted with caution due to the limited sample size. Therefore, when integrated with BMI, persistent entropy at G22 captured how irregularities in rest-activity impact pregnancy outcomes in the high BMI group.

When investigating participants with a healthy BMI (BMI <  25) in G22, complicated pregnancies were associated with higher odds of high entropy compared with uncomplicated pregnancies; however, this association did not reach statistical significance (OR = 8.14, 95% CI [0.71, 457.46]; Fisher’s exact test, p = 0.074). The wide 95% CI [0.71, 457.46], which includes the null value, highlights the imprecision of the odds ratio estimate. This suggests that the analysis was likely underpowered to detect a definitive association, potentially due to the small frequency of events observed in the 2x2 contingency table (see S5 Table in S1 text).

### 3.6 Comparison of TDA and cosinor analysis of rest-activity patterns

**Fig 13:**
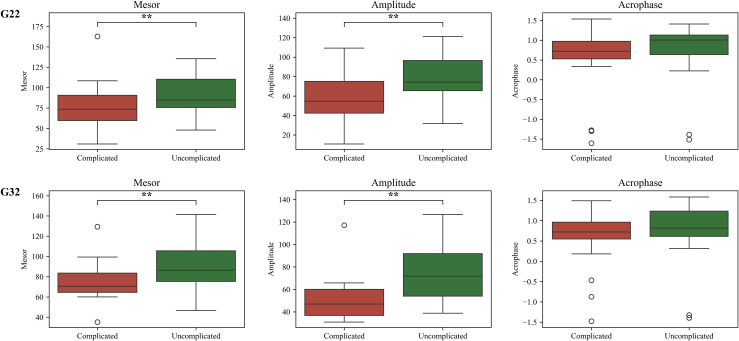
Cosinor analysis of the rest-activity cycle: Mesor, amplitude, and acrophase for participants’ rest-activity cycles in G22 and G32.

We used cosinor analysis to analyze cyclic activity and detect rhythmic patterns in the rest-activity dataset. This involves fitting a cosine function to weekly rest-activity data to determine the characteristics of rhythmic patterns, such as amplitude, MESOR, and acrophase. We compared the amplitude, MESOR, and acrophase obtained from the cosinor analysis for participants with complicated pregnancies to those with uncomplicated pregnancies. MESOR, which measures the average level around which the rest-activity data oscillates, was significantly higher for participants with uncomplicated pregnancies than for those with complicated pregnancies in both G22 and G32 (Mann-Whitney U test; G22: p = 0.04; G32: p = 0.006; Fig 13).

In addition, the amplitude, which measures the extent of oscillation or the height of the rhythmic pattern in rest-activity, was significantly higher for participants with uncomplicated pregnancies than for those with complicated pregnancies in both G22 and G32 (Mann-Whitney U test; G22: p = 0.009; G32: p = 0.0003; Fig 13). Higher amplitudes reflect more pronounced peaks and troughs in the rest-activity rhythm. There was no significant difference in the acrophase of rest-activity (i.e., the timing of the rest-activity peaks within each cycle) between participants with complicated and uncomplicated pregnancies in both G22 and G32 (Mann-Whitney U test; G22: p = 0.3; G32: p = 0.5; Fig 13).

## 4 Discussion

This study introduced the application of topological data analysis as a method for characterizing irregular visual patterns in rest-activity data. It contributes to research that investigate the ability of topology to describe complex temporal patterns in time series data [57,85,86]. A comparison of average daily persistent entropy values during G22 and G32 in women categorized as having complicated or uncomplicated pregnancies found significantly higher entropy in women with complicated pregnancies during G22, and a trend towards higher entropy during G32. Moreover, women categorized as having a complicated pregnancy had higher odds of exhibiting high weekly entropy compared to those with uncomplicated pregnancies; this was especially true for participants with high BMI. ROC analysis indicated that weekly entropy at G22 had acceptable predictive power for a complicated pregnancy, whereas entropy at G32 was a weaker predictor. This likely reflects that within the uncomplicated group, there was an increase in entropy between G22 and G32, indicating a natural loss of rest-activity patterns as pregnancy advances.

A high degree of variability in daily entropy during G22 was also associated with a complicated pregnancy. The degree of variability in daily entropy was higher in G32 than in G22. These findings are consistent with the body of work in the literature that shows that the third trimester of gestation often brings about more variability in daily routines and activity levels due to the physical demands of late pregnancy [87–89]. Physiological changes in pregnant women, including abdominal enlargement, anatomical alterations, hormonal shifts, low back pain and general fatigue can lead to more unpredictable rest and activity patterns [90,91]. Fluctuations in energy levels can result in periods of increased energy at certain times, while at other times, women may experience significant fatigue. Increased frequency of urination, fetal movement, leg cramps, and vivid dreams, which are more common during the third trimester, can disrupt sleep and daily routines, contributing to irregular patterns of rest and activity [88,92–94].

Weekly persistent entropy for rest-activity in G22 were in general higher for participants in the complicated than those in the uncomplicated pregnancy group. In particular, participants with persistent entropy above 8.22 in G22 were more likely to have a complicated pregnancy than women with persistent entropy below 8.22. This indicates that inconsistency in weekly rest-activity in G22 is associated with poor pregnancy outcomes. To evaluate whether weekly entropy during G22 or G32 predicts pregnancy complications, ROC curves were computed. Weekly entropy at G22 demonstrated acceptable predictive performance, flagging most complicated pregnancies while misclassifying a few healthy ones (AUC = 0.70; threshold = 8.24; sensitivity = 0.86; specificity = 0.70). In contrast, entropy at G32 was a weaker predictor (AUC = 0.59; threshold = 8.25; sensitivity = 0.67; specificity = 0.65). When integrated with pre-pregnancy BMI, persistent entropy at G22 captured how irregularities in rest-activity impact pregnancy outcomes. For participants with high pre-pregnancy BMI, persistent entropy above 8.22 at G22 was associated with higher odds of a complicated pregnancy compared to those below 8.22. These results suggest that irregular patterns of rest-activity at G22 may influence the odds of pregnancy complications if pre-pregnancy BMI is high. Therefore, a low entropy and a healthy pre-pregnancy BMI is important for maternal and neonatal health.

Although cosinor analysis is less rigorous and comprehensive in its description of the rest-activity data due to fragmentation in the rhythms, we found that the MESOR and amplitude provided valuable information on how rest-activity in complicated pregnancies differ from those in uncomplicated pregnancies. By combining findings from cosinor analysis with TDA, we gained a better understanding of how rest-activity patterns influence maternal and neonate health. Analysis of the MESOR and amplitude data reveals that on average individuals with uncomplicated pregnancies exhibit higher levels of physical activity compared to those with complicated pregnancies. We would like to emphasize that persistent entropy captures the degree of inconsistency in the shape of the entire rest-activity, which is different from the information obtained from the cosinor analysis. A major advantage of TDA over existing methods is that it evaluates inconsistencies in rest-activity over a given period by analyzing the shape of the entire dataset and provides a more rigorous and comprehensive description of the rest-activity pattern.

The implication of the results from this study is that we may be able to predict if a pregnancy would be complicated by evaluating (i) the level of activity at G22 obtained from cosinor analysis, (ii) the weekly disorder in rest-activity measured by the persistent entropy at G22 or (iii) the average level of daily disorder in rest-activity measured by the persistent entropy at G22. This underscores the impact of both the level of activity and the degree of disorder in rest-activity on pregnancy outcomes. The ability to predict pregnancy outcomes from rest-activity data has the potential to significantly aid clinical decision-making in maternal and neonatal care. Traditionally, treatment of complicated pregnancies usually begins after onset of disease symptoms. The findings from this study have great potential to enhance maternal and neonatal care, as they suggest that clinicians may be able to identify women at higher risk for complications such as gestational diabetes, preeclampsia, or preterm birth, earlier than traditional assessments allow. This would enable proactive interventions and timely referral to specialist care where needed. Also, the ability to interpret rest-activity data would be very useful for the implementation of individualized recommendations, thereby enhancing personalized medicine.

A limitation of using persistent entropy as a predictive variable is that the implementation of the persistent homology algorithm is often computationally intensive. This is because persistent homology involves calculating a large number of simplicial complexes for a range of parameter values, and the number of nodes in these complexes increases steeply with the size of the data [95]. If the data is too large, it may be necessary to find ways to make it more manageable. Greedy resampling was used to reduce the data size and computational cost for computing the persistence homology for each participant. Using greedy resampling, we significantly reduced the average time for computing each of the participants’ persistence homology. When we attempted to compute the weekly persistent entropy for a single participant without using greedy permutation, the computer ran out of memory after 12 hours and crashed. The computer used for the study was a MacBook Air with an Apple M1 chip, 4 cores, and 8GB of memory. It was only after using greedy permutation that we were able to compute the weekly persistent entropy for each participant in a few minutes.

Activities and health disorders that disrupt sleep quality during gestation have been found to increase the risk of delivering low birth weight infants and preterm birth and the risk of preeclampsia, hypertensive disorders and gestational diabetes mellitus [25,96,97]. Some studies have found that physical activity during gestation has a preventative property for pregnancy complications such as gestational diabetes, hypertensive disorders and weight gain [98]. Findings from our study underscore the importance of examining the cyclic patterns of rest-activity in relation to maternal and neonate health. Further study on a larger sample size is necessary to explore its use as a predictive model for maternal and neonate health outcomes. Future work may benefit from identifying an optimal rest-activity pattern that yields favorable maternal and neonatal health outcomes. Additionally, future studies may focus on how disruptions in rest-activity can negatively affect metabolic and hormonal systems during gestation, leading to unfavorable maternal and neonatal health outcomes. Furthermore, this method may be used to explore altered sleep-wake patterns in various medical disorders, both during and outside of the gestational period.

## 5 Conclusions

This study addresses a significant research gap, as the impact of day-to-day consistency in rest-activity cycles on maternal-neonate health outcomes remain poorly characterized. A key finding from this study is that TDA may be used to effectively link inconsistencies and temporal disorder in rest-activity to maternal and neonate health. It suggests a significant association between the persistent entropy of rest-activity and the occurrence of complicated pregnancies, and underscores the potential of using TDA to predict health outcomes. Findings from this study support the potential use of persistent entropy to extract patterns in rest-activity data that are predictive of maternal and neonate health outcomes. Testing this approach in a large sample size and across other populations is essential to confirm its effectiveness, determine its generalizability, and validate its efficacy in informing clinical decisions related to maternal and neonate health.

## Supporting information

S1 Text Participant characteristics and pregnancy complications. (PDF)
