# Supplementary material for: Persistent entropy links irregularities in daily and weekly rest and activity cycles during gestation week 22 and 32 to maternal and neonate health outcomes: A prospective cohort study
Source: PLoS One. 2026 Mar 16;21(3):e0342509. doi: 10.1371/journal.pone.0342509 (PMC12991255; doi:10.1371/journal.pone.0342509)
Supplement: S1 Text — (PDF) [file pone.0342509.s001.pdf]

S1 Text: Supporting Information

In this supplement, we provide additional details about the study. Table S.1 shows the study demographics including race, income, education level and average BMI for participants in complicated versus uncomplicated groups. Table S.2 shows a list of the pregnancy complications experienced by the participants and their neonates. Table S.3 shows the Fisher's Exact Test used to determine if high entropy for the whole G22 rest activity was associated with complicated/uncomplicated pregnancy outcomes. Table S.4 - S.5 shows the Fisher's Exact Test that was used to determine if high entropy was associated with BMI in G22.

Table S.1. Participant characteristics and BMI stratified by pregnancy outcome (n = 45)

**Characteristics**	**Overall** ***n(%)***	**Complicated** ***n(%)***	**Noncomplicated** ***n(%)***
**Race:**			
Black or African American	29 (64.44)	17 (77.27)	12 (52.17)
White	8 (17.77)	1 (4.54)	7 (30.43)
Asian	1 (2.22)	0 (0)	1 (4.34)
More Than One Race	5 (11.11)	3 (13.63)	2 (8.69)
Unknown	2 (4.44)	1 (4.54)	1 (4.34)
**Salary:**			
Less Than $10,000	19 (42.22)	8 (36.36)	11 (47.82)
$10,000-24,999	10 (22.22)	6 (27.27)	4 (14.39)
$25,000-49,999	10 (22.22)	7 (31.27	3 (13.04)
$50,000-74,999	2 (4.44)	1 (4.54)	1 (4.34)
Greater Than $74,000	2 (4.44)	0 (0)	2 (8.69)
Unknown	2 (4.44)	0 (0)	2 (8.69)
**Education Level:**			
Less Than 7th Grade	1 (2.22)	0 (0)	1 (4.34)
9th Grade	1 (2.22)	0 (0)	1 (4.34)
High School Graduate or GED	29 (64.44)	14 (63.63)	15 (65.21)
Bachelor's Degree	5 (11.11)	5 (22.72)	0 (0)
Associate's Degree	3 (6.66)	2 (9.09)	1 (4.34)
Graduate Degree	6 (13.33)	1 (4.54)	5 (21.73)
**BMI:**	Mean ± SD	Mean ± SD	Mean ± SD
	26.75 ± 7.67	28.55 ± 8.92	25.03 ± 5.95
**Pregnancy Outcome**			
Complicated	22 (48.88)		
Noncomplicated	23 (51.11)		

Table S.2. List of the pregnancy complications experienced by the study participants

Participant ID	Status	Neonate Complications	Maternal Complications
w3	Healthy	None	None
w4	Healthy	None	None
w5	Healthy	None	None
w6	Unhealthy	Disease	Hypertension, Preeclampsia
w8	Unhealthy	Abnormal Birth weight, Birth Length, Age at Discharge, and Gestational Age at Birth	None
w9	Healthy	None	None
w10	Unhealthy	Disease and Abnormal Birth weight, Birth Length and Gestational Age at Birth	Diabetes, Hypertension, Preeclampsia
w12	Unhealthy	Disease and Abnormal Gestational Age at Birth and Age at Discharge	Diabetes, Hypertension
w13	Healthy	None	None
w14	Unhealthy	Disease and Abnormal Gestation Age at Birth	Hypertension
w15	Unhealthy	Disease and Abnormal Gestation Age at Birth	Diabetes
w16	Unhealthy	Abnormal Gestation Age at Birth and Age at Discharge	None
w17	Unhealthy	Abnormal Age at Discharge	None
w21	Healthy	None	None
w23	Healthy	None	None
w24	Healthy	None	None
w25	Healthy	None	None
w26	Unhealthy	Abnormal Birth weight, Birth Length, Gestation Age at Birth and Age at Discharge,	None
w32	Unhealthy	Abnormal Gestation Age at Birth and Age at Discharge	None
w33	Unhealthy	Disease and Abnormal Birth Weight, Birth Length and Gestation Age at Birth	Hypertension
w36	Unhealthy	Disease and Abnormal Gestation Age at Birth	Hypertension
w38	Unhealthy	Abnormal Gestation Age at Birth	None
w39	Healthy	None	None
w41	Unhealthy	Disease and Abnormal Birth Length, Gestation Age at Birth and Age at Discharge	Diabetes, Preeclampsia
w42	Healthy	None	None
w44	Healthy	None	None
w46	Healthy	None	None
w53	Unhealthy	Abnormal Gestation Age at Birth	None
w54	Unhealthy	Disease, Abnormal Birth weight, Birth Length, Gestation Age at Birth and Age at Discharge	Hypertension
w57	Healthy	None	None
w58	Healthy	None	None
w60	Unhealthy	Disease and Abnormal Gestation Age at Birth	Hypertension
w61	Unhealthy	Abnormal Birth weight, Birth Length, Gestation Age at Birth and Age at Discharge	None
w66	Healthy	None	None
w71	Unhealthy	Disease and Abnormal Gestation Age at Birth	Preeclampsia
w75	Unhealthy	Disease and Abnormal Gestation Age at Birth	Hypertension
w76	Healthy	None	None
w79	Healthy	None	None
w81	Healthy	None	None
w82	Unhealthy	Disease and Abnormal Gestation Age at Birth	Hypertension
w83	Healthy	None	None
w84	Healthy	None	None
w85	Healthy	None	None
w87	Unhealthy	Disease and Abnormal Gestation Age at Birth	Hypertension, Preeclampsia
w93	Healthy	None	None

Table S.3. Fisher's Exact Test for G22

	High Entropy Value	Low Entropy Value	Total
Uncomplicated Pregnancy	7	13	20
Complicated Pregnancy	18	3	21
Total	25	16	41

P-value: 0.0013

Table S.4. Fisher's Exact Test for G22: High BMI (BMI > 25)

	High Entropy Value	Low Entropy Value	Total
Uncomplicated Pregnancy	1	7	8
Complicated Pregnancy	9	2	11
Total	10	9	19

P-value: 0.0055

Table S.5. Fisher's Exact Test for G22: Low BMI (BMI < 25)

	High Entropy Value	Low Entropy Value	Total
Uncomplicated Pregnancy	6	6	12
Complicated Pregnancy	9	1	10
Total	15	7	22

P-value: 0.0743
